# Supplementary material for: HPLC–(Q)-TOF-MS-Based Study of Plasma Metabolic Profile Differences Associated with Age in Pediatric Population Using an Animal Model
Source: Metabolites. 2022 Aug 11;12(8):739. doi: 10.3390/metabo12080739 (PMC9413543; doi:10.3390/metabo12080739)
Supplement: Supplementary file 1 [file metabolites-12-00739-s001.zip › metabolites-1839550-supplementary.pdf]

Supplementary Material

Article

HPLC-(Q)-TOF-MS based study of plasma metabolic profiles differences associated with age in paediatric population using animal model

Oihane E. Albóniga[1,2*], Oskar González-Mendia[3], María E. Blanco[1], Rosa M. Alonso[1]

[1]Department of Analytical Chemistry, Faculty of Science and Technology, University of the Basque Country (UPV/EHU), Barrio Sarriena s/n, 48940 Leioa, Spain
[2] Metabolomics Platform, CIC bioGUNE, CIBERehd, Bizkaia Technology Park, 48160, Bilbao, Spain.
[3]Painting Department, Faculty of Fine Arts, University of the Basque Country (UPV/EHU), Barrio Sar-riena s/n, 48940 Leioa, Spain
*Correspondence: oihaneelena.alboniga@ehu.eus

Contents:

Figure S1. PCA scores plot obtained after scaling the matrices by autoscaling for Plasma ESI+ and logarithm for Plasma ESI-, and PCA scores plot after QC correction. Piglet groups: newborns (A), infants (B), and children (C). TEV = total explained variance.

Figure S2. Plasma ESI+ validation results. **a.** Average confusion matrix in percentage and **b.** Correct classification rate (CCR) graph where blue bars are the real sample classification and the red bars the permuted or randomly assigned classification.

Figure S3. Plasma ESI- validation results. **a.** Average confusion matrix in percentage and **b.** Correct classification rate (CCR) graph where blue bars are the real sample classification and the red bars the permuted or randomly assigned classification.

Table S1. Pairs of correlated features obtained by Pearson correlation studies that were considered for MS/MS studies.

Figure S4. Experimental fragmentation pattern of the significant feature in positive ionization mode with m/z = 548.3703 and RT = 11.52 min, which has a phosphocholine in its structure.

Figure S5. Experimental fragmentation pattern of the significant feature in positive ionization mode with m/z = 300.6346 and RT = 10.55 min, which has a phosphocholine in its structure.

Figure S6. Fragmentation pattern of the significant feature with m/z = 356.2794 and RT = 9.7 annotated as acylcarnitine class.

Figure S7. MS/MS spectra and fragmentation pattern in negative ionization mode for the LPC (20:5) with m/z = 586.3141 and RT = 10.03 min.

Figure S8. MS/MS spectra and fragmentation pattern in negative ionization mode for the LPC class metabolite with m/z = 615.3475 and RT = 10.69 min.

Figure S9. MS/MS spectra and fragmentation pattern for the LPE (18:1) with m/z = 478.2922 and RT = 10.89 min.

Figure S10. MS/MS spectra and fragmentation pattern in negative ionization mode for the LPE (15:1) with m/z = 436.2815 and RT = 10.98 min.

Figure S11. Tendency among groups of the significant metabolites (A) LCP (20:4) obtained in positive ionization mode and (B) LPC (20:5) in negative ionization mode.

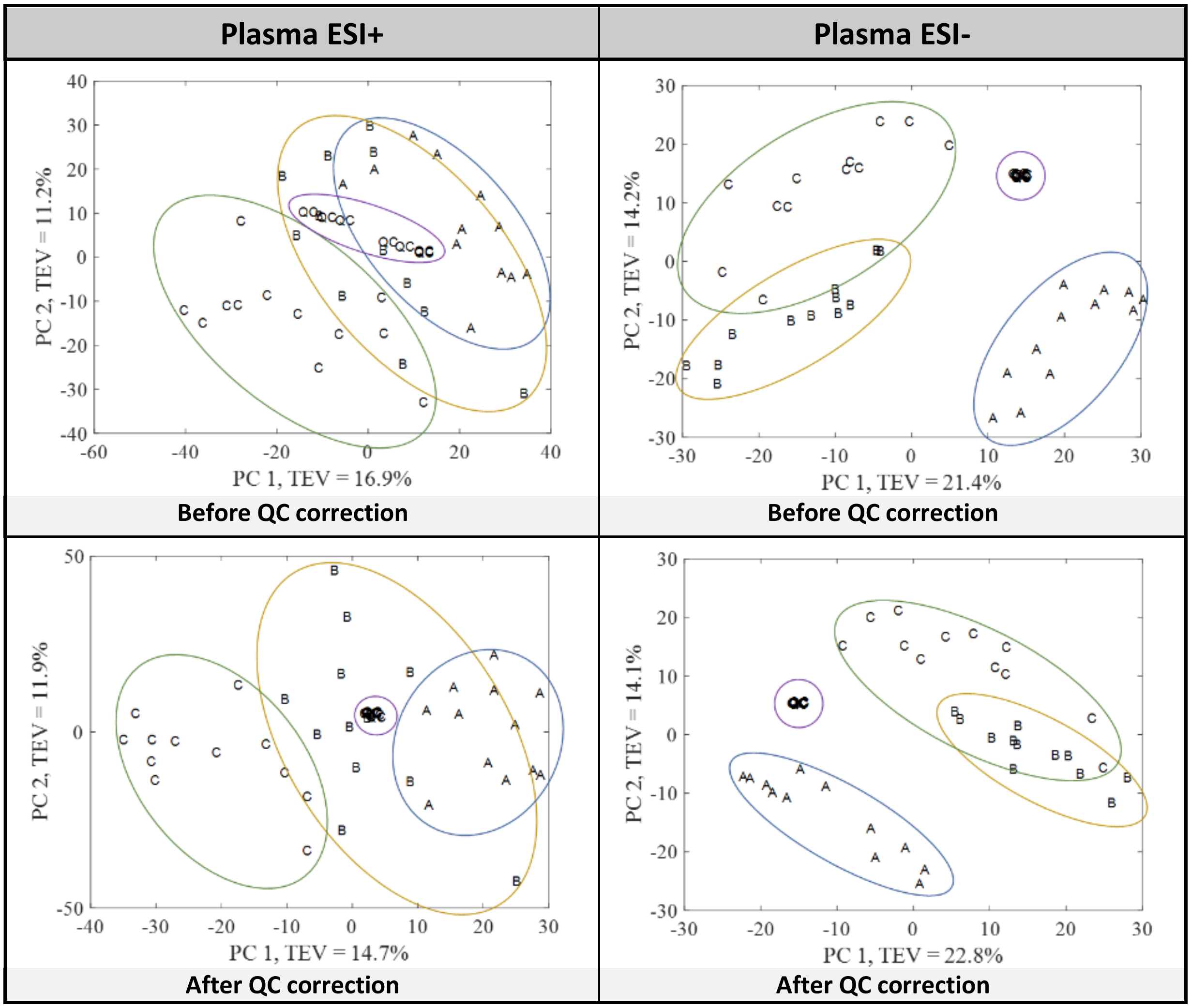

Figure S1. PCA scores plot obtained after scaling the matrices by autoscaling for Plasma ESI+ and logarithm for Plasma ESI-, and PCA scores plot after QC correction. Piglet groups: newborns (A), infants (B), and children (C). TEV = total explained variance.

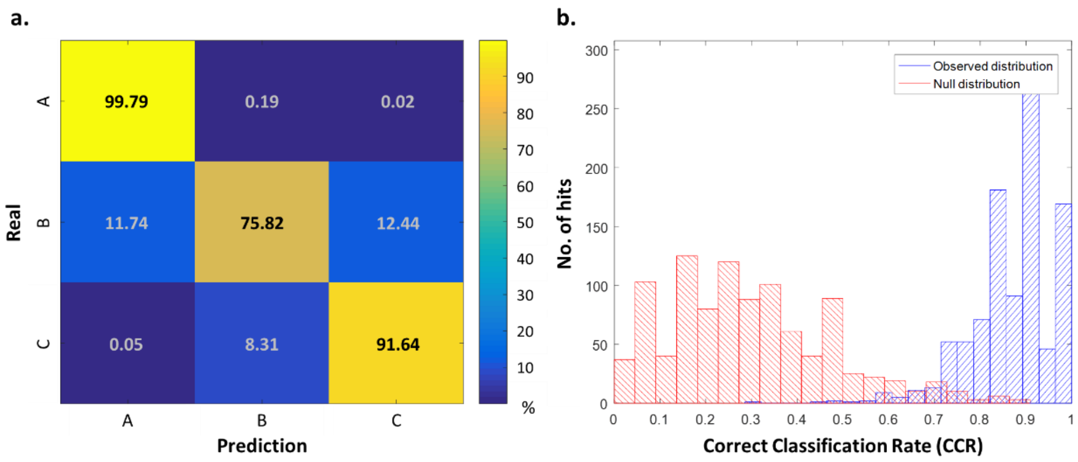

Figure S2. Plasma ESI+ validation results. **a.** Average confusion matrix in percentage and **b.** Correct classification rate (CCR) graph where blue bars are the real sample classification and the red bars the permuted or randomly assigned classification.

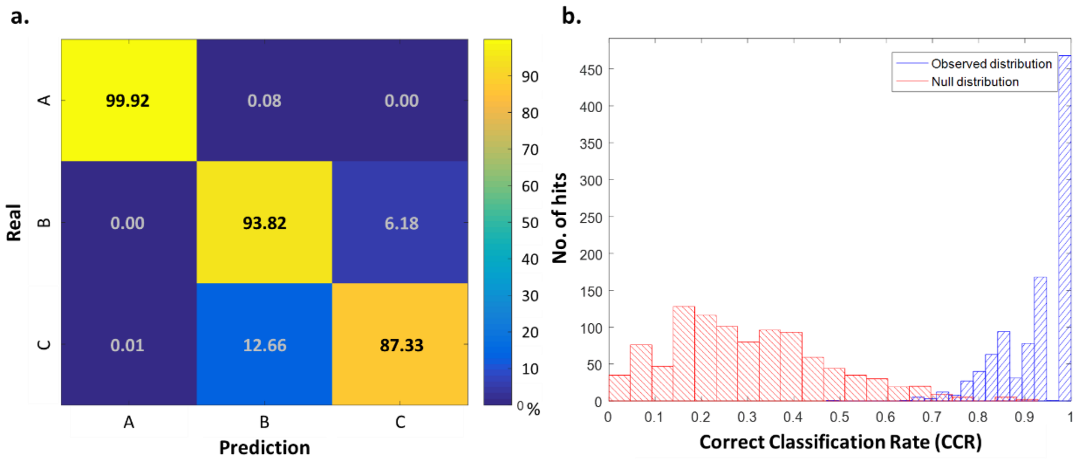

Figure S3. Plasma ESI- validation results. **a.** Average confusion matrix in percentage and **b.** Correct classification rate (CCR) graph where blue bars are the real sample classification and the red bars the permuted or randomly assigned classification.

Table S1. Pairs of correlated features obtained by Pearson correlation studies that were considered for MS/MS studies.

m/z	RT (min)	Putative Ion Specie	Ionization Mode	*m/z*	RT (min)	Putative Ion Specie	Ionization Mode
400.1157*	6.57	$[M+H]^+$	ESI+	398.0972*	6.52	$[M-H]^-$	ESI-
271.9848*	6.84		ESI+	343.0242*	6.78		ESI-
				457.0161*	6.79		ESI-
364.0715*	6.98		ESI+	351.0658	6.96		ESI-
530.3254*	11.04	$[M+H]^+$	ESI+	528.3077	11.01	$[M-H]^-$	ESI-
237.0881	6.10	$[M+H]^+$	ESI+	235.0707*	5.95	$[M-H]^-$	ESI-
309.0737	6.41		ESI+	315.1055*	6.37		ESI-
146.0599	6.70		ESI+	230.0111*	6.66		ESI-
430.1264	7.85	$[M+H]^+$	ESI+	428.1105*	7.83	$[M-H]^-$	ESI-
243.6238	9.17		ESI+				
244.6377	9.11		ESI+				
355.2625	9.11		ESI+				
373.2735	9.10		ESI+	815.5669*	9.11	$[M-H]^-$	ESI-
391.2836	9.11		ESI+				
493.2501	9.10		ESI+				
839.5638	9.11	$[M+Na]^+$	ESI+				

* Significant features that differentiate the three groups (A≠B≠C)

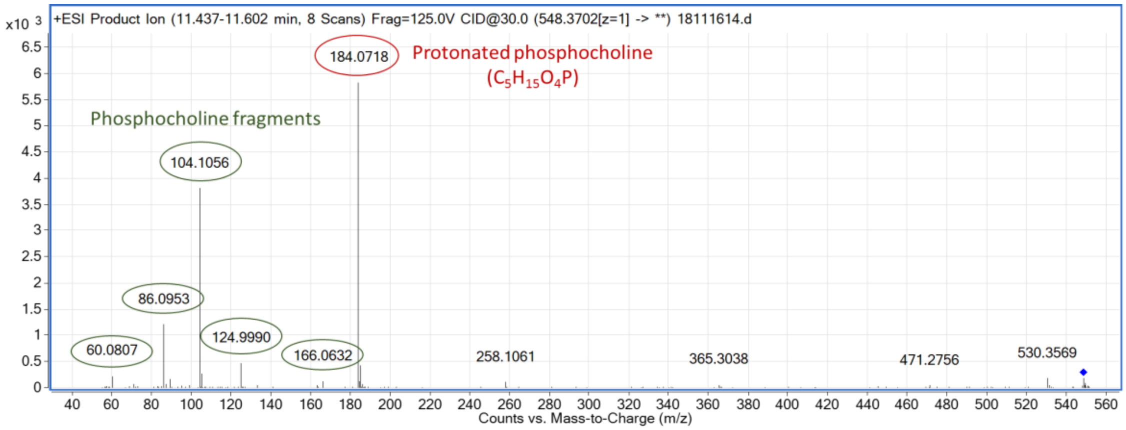

Figure S4. Experimental fragmentation pattern of the significant feature in positive ionization mode with m/z = 548.3703 and RT = 11.52 min, which has a phosphocholine in its structure.

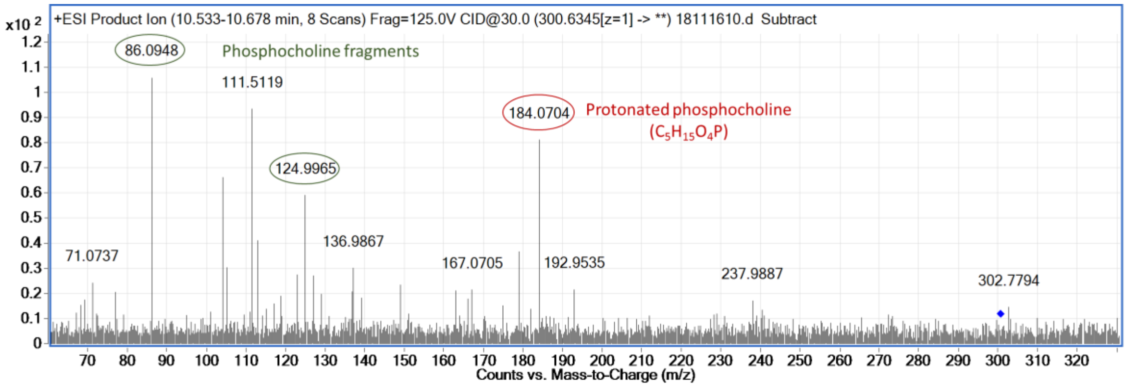

Figure S5. Experimental fragmentation pattern of the significant feature in positive ionization mode with m/z = 300.6346 and RT = 10.55 min, which has a phosphocholine in its structure.

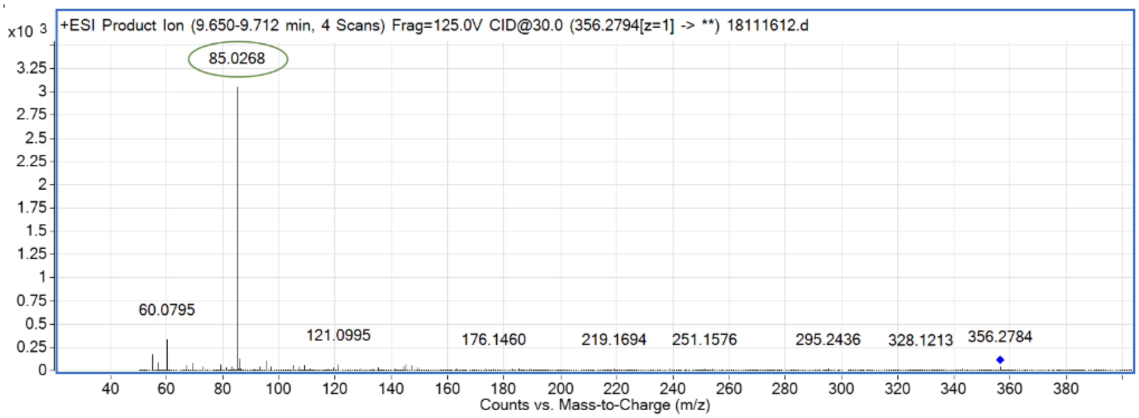

Figure S6. Fragmentation pattern of the significant feature with m/z = 356.2794 and RT = 9.7 annotated as acylcarnitine class.

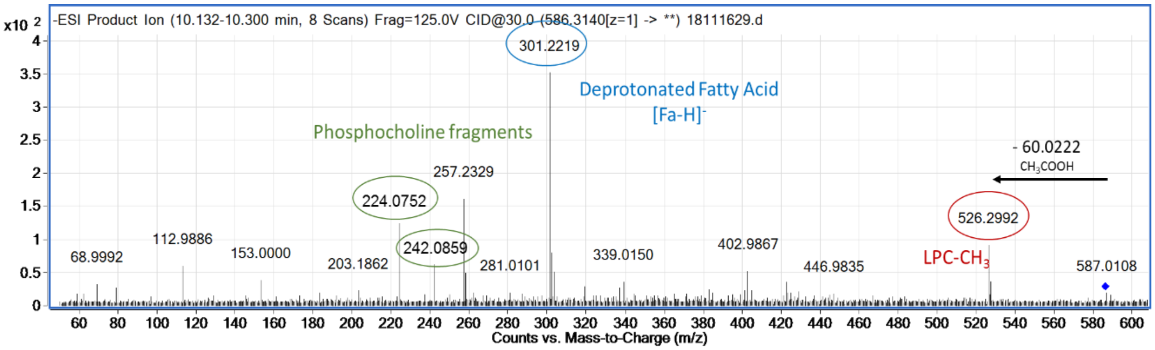

Figure S7. MS/MS spectra and fragmentation pattern in negative ionization mode for the LPC (20:5) with m/z = 586.3141 and RT = 10.03 min.

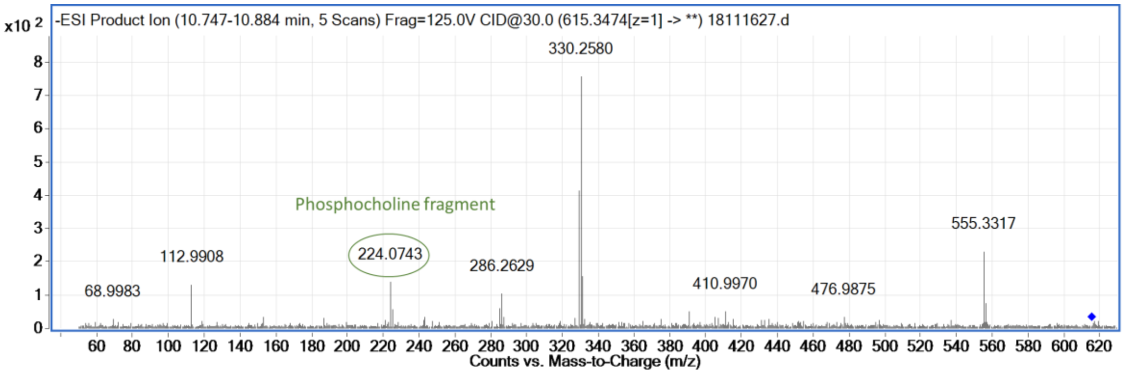

Figure S8. MS/MS spectra and fragmentation pattern in negative ionization mode for the LPC class metabolite with m/z = 615.3475 and RT = 10.69 min.

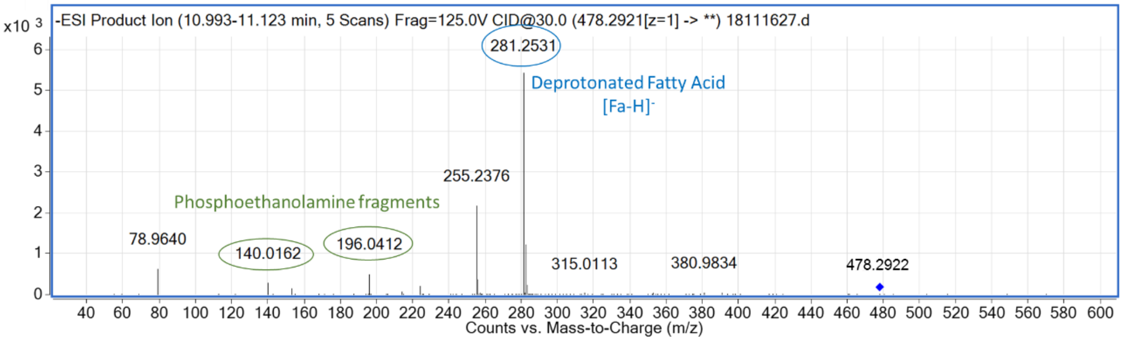

Figure S9. MS/MS spectra and fragmentation pattern for the LPE (18:1) with m/z = 478.2922 and RT = 10.89 min.

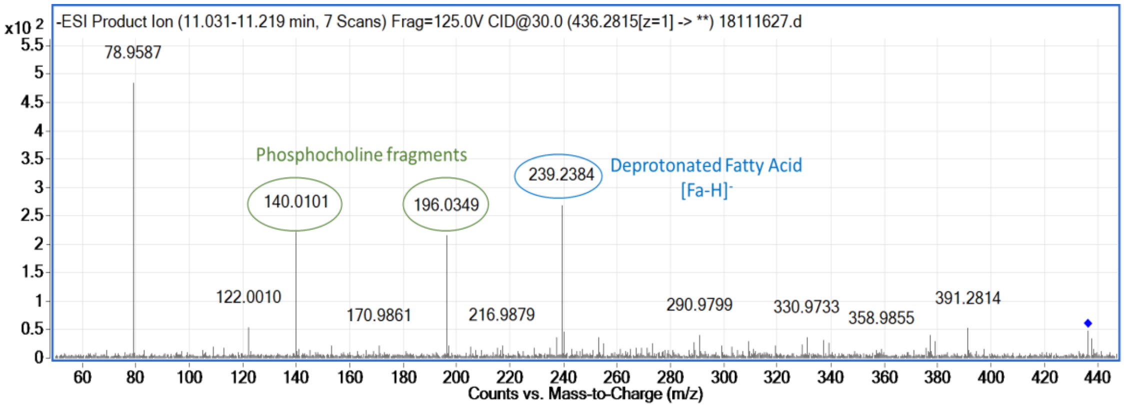

Figure S10. MS/MS spectra and fragmentation pattern in negative ionization mode for the LPE (15:1) with m/z = 436.2815 and RT = 10.98 min.

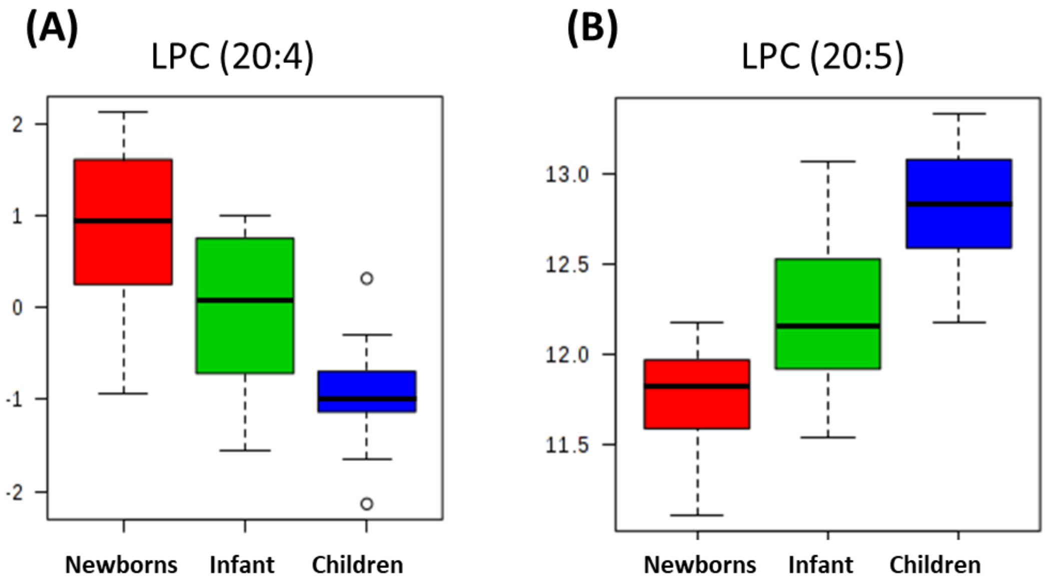

Figure S11. Tendency among groups of the significant metabolites (A) LCP (20:4) obtained in positive ionization mode and (B) LPC (20:5) in negative ionization mode.
